# Use of Aspirin for Prevention of Recurrent Atherosclerotic Cardiovascular Disease Among Adults — 20 States and the District of Columbia, 2013

**Published:** 2015-07-17

**Authors:** Jing Fang, Mary G. George, Yuling Hong, Fleetwood Loustalot

**Affiliations:** 1 Division for Heart Disease and Stroke Prevention, National Center for Chronic Disease Prevention and Health Promotion, CDC

The effectiveness of regular aspirin therapy in reducing risk (secondary prevention) for myocardial infarction, ischemic stroke, and fatal coronary events among persons with preexisting atherosclerotic cardiovascular disease (ASCVD) is well established (1) and recommended in current guidelines (2). Reported use of aspirin or other antiplatelet agents for secondary ASCVD prevention has varied widely across settings and data collection methods, from 54% of outpatient visits for those with ischemic vascular disease (3) to 98% at the time of discharge for acute coronary syndrome (4). To estimate the prevalence of aspirin use for secondary ASCVD prevention among community-dwelling adults, CDC analyzed 2013 Behavioral Risk Factor Surveillance System (BRFSS) data from 20 states and the District of Columbia. Overall, 70.8% of adult respondents with existing ASCVD reported using aspirin regularly (every day or every other day). Within this group, 93.6% reported using aspirin for heart attack prevention, 79.6% for stroke prevention and 76.2% for both heart attack and stroke prevention. Differences in use were found by age, sex, race/ethnicity, and ASCVD risk status, and state. Most of the state differences were not statistically significant; however, these estimates can be used to promote the use of aspirin as a low-cost (2) and highly effective intervention (1).

BRFSS is an annual telephone survey, conducted by all U.S. states, with guidance and support from CDC. Detailed information regarding the survey can be found online (at http://www.cdc.gov/brfss ). In addition to core questions asked by all states, optional BRFSS modules are dedicated to various topic areas. In 2013, 20 states (Arizona, Arkansas, Florida, Georgia, Hawaii, Iowa, Maine, Massachusetts, Minnesota, Mississippi, Missouri, Nebraska, North Carolina, North Dakota, Oklahoma, Oregon, South Carolina, Tennessee, Washington, and Wisconsin) and the District of Columbia opted to include the cardiovascular health module in their surveys. This module included questions about regular (every day or every other day) aspirin use. Respondents who answered “no” when asked about regular aspirin use were subsequently asked whether they had any health problem or condition that made taking aspirin unsafe (e.g., a “stomach condition”). Those who answered “yes,” to regular aspirin use were asked additional questions to learn the reason for aspirin use (i.e., to reduce the chance of heart attack, to reduce the chance of stroke, and to relieve pain).*

Participants were classified as having preexisting ASCVD if they reported a history of coronary heart disease or stroke, based on their answers to the following questions: “Has a doctor, nurse, or other health professional ever told you that you had 1) a heart attack, also called a myocardial infarction; 2) angina or coronary heart disease; or 3) a stroke?” Self-reported sociodemographic and descriptive characteristics included age, sex, race/ethnicity, education, and selected ASCVD risk factors (hypertension, diabetes, high cholesterol, current smoking). Prevalence of regular aspirin use was estimated only among those with preexisting ASCVD, stratified by sociodemographic characteristics and number of ASCVD risk factors. Age-standardized prevalence of aspirin use among persons with ASCVD by state was estimated using the 2000 U.S. standard population, based on the following age groups: 18–24, 25–44, 45–64 and ≥65 years (5).

Overall, 175,523 participants aged ≥18 years from 20 states and the District of Columbia responded to the cardiovascular health module, and 21,682 (12.5%) reported a history of coronary heart disease, stroke, or both. From this group, 3,698 (17.1%) were excluded because of missing sociodemographic and ASCVD risk factor data, resulting in a final analytic sample of 17,984. The number of participants ranged from 387 (District of Columbia) to 4,227 (Florida). The median state response rate, calculated according to guidelines of the American Association of Public Opinion Research, was 44%; response rates among states and the District of Columbia ranged from 31% to 59% (6).

Among the eligible respondents with preexisting ASCVD, 70.8% reported regular aspirin use, with 93.6% taking it to prevent a heart attack, 79.6% to prevent a stroke, and 76.2% to prevent both heart attack and stroke (Table 1). Moreover, 14.9% (95% confidence interval [CI]: 13.8%–16.0%) of eligible respondents who reported regular aspirin use and who had ASCVD also reported using aspirin for pain relief, and 4.2% (CI: 3.5%–4.9%) of eligible respondents reported using aspirin for pain relief only. The percentage of aspirin use for prevention of secondary of ASCVD varied by sociodemographic characteristics (Table 1). In general, respondents aged ≥65 years, men, non-Hispanic whites and those with at least two ASCVD risk factors were more likely to use aspirin than other groups.

By state, the age-standardized percentage of regular aspirin use among those with ASCVD ranged from 44.3% (Missouri) to 71.7% (Mississippi) (Table 2) with wide confidence intervals, and most of the observed differences among the states were not statistically significant. No systematic pattern of aspirin use by region was observed (Figure).

## Discussion

Overall, 70.8% of adults with preexisting ASCVD in 20 states and the District of Columbia reported regular aspirin use, but differences in aspirin use among various groups were found. Aspirin use for the prevention of recurrent ASCVD is widely promoted across the United States, and is included in national cardiovascular disease prevention programs such as the Million Hearts initiative (7) and *Healthy People 2020* (8). Furthermore, CDC-funded state programs to prevent and control heart disease and stroke (e.g., State Public Health Actions to Prevent and Control Diabetes, Heart Disease, Obesity and Associated Risk Factors and Promote School Health† and Well-Integrated Screening and Evaluation for Women Across the Nation [WISEWOMAN]§) support states using strategies for cardiovascular disease and risk factors management outlined in the Million Hearts initiative. These include promotion of the “ABCS” of clinical prevention: aspirin use when appropriate, blood pressure control, cholesterol management, and smoking cessation, as well as promoting healthy environments and encouraging a heart-healthy lifestyle.

Although the overall self-reported prevalence of aspirin use among persons with ASCVD in the BRFSS cardiovascular health module was similar to the 70% recently reported for the 2012 National Health Interview Survey (9), variations in aspirin use were observed in this analysis among geographic areas and sociodemographic groups. Public health practitioners and clinicians can use data from this report to target resources and interventions to those groups with lower use of aspirin. Promotion of adherence to evidence-based practice guidelines and clinical management algorithms upon discharge after cardiovascular disease events, counseling about aspirin use, and implementation of community-based interventions to champion the benefits of regular aspirin use among those eligible are needed to increase aspirin use (10). Further work is needed to assess possible variation in aspirin use at subnational levels and among different risk groups. Although regular aspirin use for pain relief among adults with ASCVD was uncommon, 4.2% of those with ASCVD were using it for pain relief only and not for prevention of heart attack or stroke, receiving the therapeutic benefit of aspirin for secondary ASCVD prevention without realizing it.

The findings in this report are subject to at least five limitations. First, current guidelines recommend aspirin or other antiplatelet medications for prevention of recurrent events; however, data on the use of other antiplatelet medications (as alternatives to aspirin) was not collected by BRFSS. Therefore, the overall use of all antiplatelet medications could not be estimated. Second, the use of aspirin is generally contraindicated after a hemorrhagic stroke (intracerebral hemorrhage and subarachnoid hemorrhage), and BRFSS did not distinguish between hemorrhagic and ischemic stroke. This may partially account for the lower reported prevalence of regular aspirin use for stroke prevention compared with that for heart attack prevention; however, hemorrhagic stroke accounts only for about 10% of all strokes (4). Third, these data are self-reported, and aspirin does not require a prescription for purchase, which might lead to recall bias as well as challenges in identifying the medication. Fourth, because not all states participated in this BRFSS module, the data are not nationally representative. Finally, with relatively small samples of respondents with coronary heart disease or stroke at the state level, confidence intervals for state level estimates were wide.

Although provision of aspirin at discharge following a cardiovascular disease event is high (4), reports using community-based data sources find that aspirin use for secondary prevention is suboptimal. Consistent and timely access to health care services encourages the assessment of ASCVD by clinicians, and community-based interventions to promote aspirin use might reach those persons not likely to interact with health care providers on a regular basis. In addition, interventions targeting specific subgroups, such as those younger than age 65 years, women, and black and Hispanic patients might reduce disparities in aspirin use. The use of this low-cost, effective, and generally safe intervention among persons who have existing atherosclerotic cardiovascular disease is supported by multiple evidence-based guidelines, and current data suggest that there is room for increased use in this population.

**Summary**

What is already known on this topic?

Aspirin and other antiplatelet medications reduce the risk of cardiovascular events among adults with existing atherosclerotic cardiovascular disease (ASCVD).

What is added by this report?

Among persons with preexisting ASCVD in 20 US states and the District of Columbia, 70.8% reported using aspirin regularly for prevention of heart attack and stroke, and differences were observed in reported aspirin use among certain groups.

What are the implications for public health practice?

Clinical and community-based approaches should be used to increase aspirin use among persons with ASCVD to prevent recurrent heart attacks and strokes, with specific attention to groups reporting lower aspirin use.

## Figures and Tables

**FIGURE:**
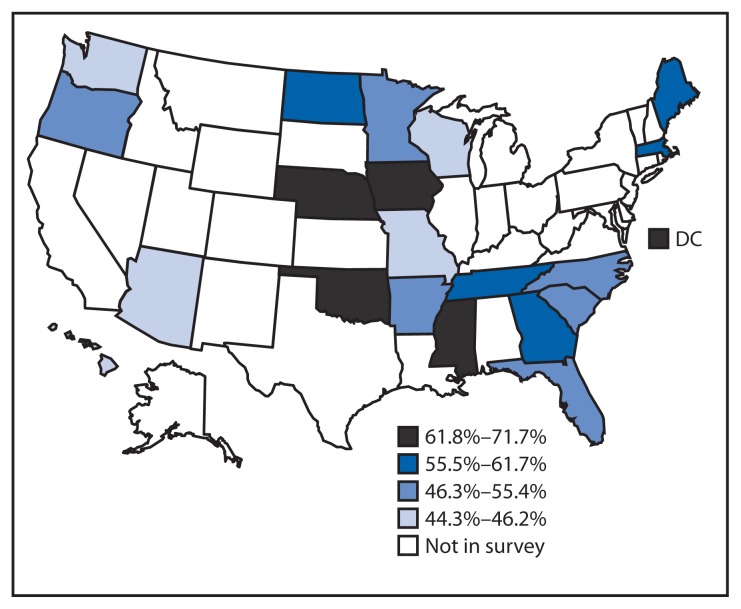
Age-standardized percentage of aspirin use among persons with preexisting atherosclerotic cardiovascular disease, by quartile — Behavioral Risk Factor Surveillance System — 20 states and the District of Columbia (DC), 2013

**TABLE 1:** Age-standardized percentage* of adults taking aspirin for secondary prevention of atherosclerotic cardiovascular disease and reason for taking aspirin, by selected characteristics — Behavioral Risk Factor Surveillance System, 20 states and the District of Columbia, 2013

				Among those using aspirin regularly,† they used it to prevent					
			
		Total use of aspirin		Heart attack		Stroke		Both heart attack and stroke	
					
Characteristic	No.	%	(95% CI)	%	(95% CI)	%	(95% CI)	%	(95% CI)
**Total**	**17,984**	**70.8**	**(69.4–72.1)**	**93.6**	**(92.7–94.5)**	**79.6**	**(78.1–81.1)**	**76.2**	**(74.6–77.8)**
**Age group (yrs)**									
18–64	6,181	65.9	(63.5–68.2)	92.9	(91.2–94.3)	80.8	(78.3–83.1)	77.2	(74.5–79.6)
≥65	1,1803	75.0	(73.5–76.5)	94.2	(93.1–95.1)	78.6	(76.6–80.4)	75.4	(73.4–77.4)
**Sex**									
Men	8,518	76.2	(74.4–77.9)	95.0	(93.9–95.9)	78.5	(76.2–80.6)	76.2	(73.9–78.3)
Women	9,466	64.4	(62.3–66.3)	91.7	(90.0–93.1)	81.2	(79.2–83.0)	76.3	(74.0–78.4)
**Race/Ethnicity** §									
White	14,595	73.6	(72.2.74.9)	94.3	(93.4–95.1)	79.6	(78.1–81.1)	76.7	(75.1–78.2)
Black	1,889	63.0	(58.2–67.6)	93.6	(90.6–95.6)	81.5	(76.6–85.6)	77.6	(72.5–82.0)
Hispanic	398	55.6	(45.7–65.1)	83.3	(70.7–91.2)	71.8	(55.4–83.9)	61.9	(46.5–76.3)
Other	1,102	64.7	(58.5–70.5)	92.7	(88.4–95.5)	81.6	(75.0–86.7)	78.4	(71.6–83.9)
**Education**									
Less than high school diploma	2,469	65.0	(61.1–68.7)	93.0	(90.2–95.0)	78.1	(72.8–82.6)	74.8	(69.5–79.4)
High school diploma	6,066	72.6	(70.3–74.7)	92.7	(90.7–94.3)	79.8	(77.5–81.9)	75.9	(73.3–78.3)
Some college	5,099	69.8	(67.4–72.1)	94.6	(93.2–95.8)	81.1	(78.4–83.5)	78.3	(75.6–80.8)
College degree	4,350	76.7	(74.2–79.0)	94.3	(92.6–95.7)	78.4	(75.6–81.0)	74.9	(71.9–77.6)
**No. of cardiovascular risk factors** ¶									
None	1,359	54.7	(49.4–59.9)	89.6	(85.5–92.7)	72.6	(65.7–78.6)	65.9	(58.9–72.2)
1	4,027	63.4	(60.3–66.4)	90.5	(87.9–92.6)	77.3	(74.2–80.2)	71.4	(67.9–74.7)
2	7,289	74.5	(72.3–76.5)	93.8	(92.2–95.2)	79.7	(77.2–82.0)	76.8	(74.2–79.2)
3	4,662	76.1	(73.7–78.5)	95.9	(84.6–97.0)	81.8	(78.7–84.6)	80.0	(76.8–82.8)
4	647	72.7	(66.2–78.4)	97.1	(94.0–98.6)	84.2	(78.3–88.7)	83.4	(77.5–88.0)

**Abbreviation:** CI = confidence interval.

* Using the U.S. 2000 standard projected population with age groups of 18–24, 25–44, 45–64 and ≥65 years.

† Every day or every other day.

§ All white, black, and other respondents were non-Hispanic. Hispanic respondents might be of any race.

¶ Hypertension, diabetes, high cholesterol, and current smoking. Risk factors were each given a weight of 1 and totaled.

**TABLE 2:** Age-standardized percentage* of adults taking aspirin for secondary prevention of atherosclerotic cardiovascular disease and reason for taking aspirin, by state — Behavioral Risk Factor Surveillance System, 20 states and the District of Columbia (DC), 2013

				Among those using aspirin regularly,† they used it to prevent					
			
		Total use of aspirin		Heart attack		Stroke		Both heart attack and stroke	
					
State/Area	No.	%	(95% CI)	%	(95% CI)	%	(95% CI)	%	(95% CI)
Arizona	437	44.6	(30.7–59.4)	86.3	(54.9–97.0)	78.8	(52.7–92.5)	75.9	(51.1–90.5)
Arkansas	647	54.9	(43.0–66.3)	89.5	(62.7–97.8)	82.6	(61.9–93.3)	81.4	(61.2–92.4)
DC	378	69.5	(55.4–80.7)	89.8	(76.4–96.0)	83.4	(71.2–91.1)	74.6	(70.0–78.8)
Florida	4227	51.2	(41.6–60.7)	89.3	(77.0–95.4)	67.7	(57.1–76.8)	65.6	(55.1–74.7)
Georgia	729	57.8	(49.0–66.1)	91.6	(77.5–97.2)	82.8	(75.6–88.2)	76.3	(64.5–85.1)
Hawaii	513	46.2	(35.0–57.8)	87.5	(67.4–96.0)	82.2	(64.5–92.1)	79.9	(62.9–90.3)
Iowa	827	71.2	(58.4–81.3)	85.6	(69.2–94.0)	69.9	(53.8–82.2)	59.2	(43.3–73.4)
Maine	497	61.7	(51.3–71.1)	95.3	(83.1–98.8)	70.5	(55.0–82.4)	66.5	(50.7–79.4)
Massachusetts	379	56.5	(35.9–75.1)	95.6	(88.6–98.4)	60.8	(38.3–79.4)	58.2	(36.0–77.6)
Minnesota	1082	55.1	(43.7–66.0)	80.9	(58.9–92.7)	81.0	(65.7–90.5)	68.8	(50.0–82.9)
Mississippi	926	71.7	(59.9–81.0)	92.1	(83.3–96.5)	82.1	(70.4–89.9)	80.5	(69.0–88.5)
Missouri	811	44.3	(35.3–53.6)	92.9	(73.8–98.4)	63.8	(38.6–83.1)	58.4	(37.7–76.5)
Nebraska	850	71.5	(59.7–80.9)	77.3	(68.0–84.5)	56.5	(41.6–70.3)	53.1	(38.4–67.2)
North Carolina	487	47.3	(38.0–56.8)	95.8	(92.4–97.8)	80.9	(62.0–91.6)	78.4	(60.2–89.7)
North Dakota	711	59.2	(45.9–71.2)	88.7	(68.5–96.6)	77.7	(53.5–91.3)	68.1	(46.4–84.1)
Oklahoma	537	67.0	(52.6–78.8)	85.6	(84.7–86.5)	69.9	(58.5–79.3)	69.3	(58.0–78.8)
Oregon	508	50.2	(36.9–63.5)	78.3	(57.6–90.5)	67.4	(48.8–81.7)	61.1	(43.6–76.1)
South Carolina	1148	55.4	(47.4–63.1)	96.5	(94.3–97.8)	89.0	(83.9–92.7)	86.9	(81.6–90.8)
Tennessee	725	58.2	(44.7–70.5)	76.8	(65.2–85.3)	76.2	(67.8–83.0)	70.8	(59.3–80.2)
Washington	1041	44.4	(37.0–52.0)	95.5	(92.7–97.3)	85.5	(78.7–90.4)	83.8	(77.1–88.9)
Wisconsin	524	45.5	(40.0–51.2)	98.3	(96.2–99.2)	87.7	(70.3–95.5)	86.4	(69.7–94.6)

**Abbreviation:** CI = confidence interval.

* Using the U.S. 2000 standard projected population with age groups of 18–24, 25–44, 45–64 and ≥65 years.

† Every day or every other day.

## Footnotes

* The questions asked were as follows: “Do you take aspirin daily or every other day?” “Do you have a health problem or condition that makes taking aspirin unsafe for you?” “Do you take aspirin to relieve pain?” “Do you take aspirin to reduce the chance of a heart attack?” “Do you take aspirin to reduce the chance of a stroke?”

† Available at http://www.cdc.gov/dhdsp/programs/spha/index.htm.

§ Available at http://www.cdc.gov/wisewoman/.
